# The Auditory-Visual Speech Benefit on Working Memory in Older Adults with Hearing Impairment

**DOI:** 10.3389/fpsyg.2016.00490

**Published:** 2016-04-12

**Authors:** Jana B. Frtusova, Natalie A. Phillips

**Affiliations:** Cognition, Aging, and Psychophysiology Lab, Department of Psychology, Concordia UniversityMontreal, QC, Canada

**Keywords:** aging, hearing impairment, speech perception, multisensory interaction, working memory, even-related potentials

## Abstract

This study examined the effect of auditory-visual (AV) speech stimuli on working memory in older adults with poorer-hearing (PH) in comparison to age- and education-matched older adults with better hearing (BH). Participants completed a working memory *n*-back task (0- to 2-back) in which sequences of digits were presented in visual-only (i.e., speech-reading), auditory-only (A-only), and AV conditions. Auditory event-related potentials (ERP) were collected to assess the relationship between perceptual and working memory processing. The behavioral results showed that both groups were faster in the AV condition in comparison to the unisensory conditions. The ERP data showed perceptual facilitation in the AV condition, in the form of reduced amplitudes and latencies of the auditory N1 and/or P1 components, in the PH group. Furthermore, a working memory ERP component, the P3, peaked earlier for both groups in the AV condition compared to the A-only condition. In general, the PH group showed a more robust AV benefit; however, the BH group showed a dose-response relationship between perceptual facilitation and working memory improvement, especially for facilitation of processing speed. Two measures, reaction time and P3 amplitude, suggested that the presence of visual speech cues may have helped the PH group to counteract the demanding auditory processing, to the level that no group differences were evident during the AV modality despite lower performance during the A-only condition. Overall, this study provides support for the theory of an integrated perceptual-cognitive system. The practical significance of these findings is also discussed.

## Introduction

Aging is associated with various physical and cognitive changes, including both structural and functional changes in the auditory system resulting in hearing difficulty. Hearing impairment is the third most common chronic condition in older adults, ranking just after arthritis, and hypertension (Zhang et al., 2013) and it has a significant impact on older adults' quality of life (e.g., Strawbridge et al., 2000; Dalton et al., 2003). The most common cause of hearing impairment in older adults results from various structural and functional age-related changes in the cochlea (Schneider, 1997). In addition to elevated hearing thresholds, these changes affect the processing of temporal and spectral cues, which are important for speech perception (e.g., Baer and Moore, 1994; Schneider, 1997; Schneider and Pichora-Fuller, 2001; Pichora-Fuller et al., 2007). Research also indicates that older adults need to engage broader cortical networks to process speech compared to younger adults (Wong et al., 2009). Thus, age-related changes in the auditory system can have a negative effect on speech perception, making it more effortful, and resource demanding.

In addition to hearing difficulty, one of the most common complaints of older adults is difficulty with remembering information. According to a model proposed by Schneider and Pichora-Fuller (2000) there is a direct link between perceptual and higher-order cognitive functioning, such as memory. More specifically, they have proposed that perceptual and cognitive functions share a common pool of processing resources. Under this theory, having to devote too many processing resources toward perception may result in insufficient residual resources for subsequent higher-order processing, such as encoding and storing of the information in memory. Thus, for older adults with hearing impairment, memory difficulty may be a secondary effect of having to devote too many processing resources to speech perception. This has been demonstrated by several studies, which have shown that hearing impairment as well as presentation of auditory information in background noise interferes with memory performance (e.g., Rabbitt, 1968, 1991; Pichora-Fuller et al., 1995; McCoy et al., 2005).

In contrast to a negative effect of hearing impairment, there is strong evidence indicating that auditory-visual (AV) speech, in which both auditory and visual speech cues (i.e., lip, tongue, and face movements) are available, enhances speech recognition (e.g., Sumby and Pollack, 1954; Klucharev et al., 2003; Bernstein and Grant, 2009; Ma et al., 2009; Tanaka et al., 2009; Fraser et al., 2010; Winneke and Phillips, 2011). Importantly, AV speech is not only associated with behavioral improvements of speech perception, but also with more efficient brain processing. This effect is indicated by studies using event-related potential (ERP) methodology, which measures electrical brain activity associated with different stages of stimulus-related processing (Luck, 2005). ERP components relevant to speech perception include the P1, which refers to a positive-going waveform peaking approximately 50 ms after the onset of the stimulus and that is proposed to originate from the primary auditory cortex (Liegeois-Chauvel et al., 1994), and the N1, which is a negative-going waveform that peaks approximately 100 ms after the onset of a sound and is proposed to originate from the secondary auditory cortex (Liegeois-Chauvel et al., 1994; Pantev et al., 1995).

The data from ERP research suggest that the brain elicits earlier and smaller responses during AV speech in comparison to auditory-only (A-only) speech modality. More specifically, both amplitude (van Wassenhove et al., 2005; Stekelenburg and Vroomen, 2007; Frtusova et al., 2013) and latency (van Wassenhove et al., 2005; Stekelenburg and Vroomen, 2007; Pilling, 2009; Winneke and Phillips, 2011; Frtusova et al., 2013) of the auditory N1 and/or P1 component are reduced during processing of AV compared to A-only speech. Overall, these results indicate that the brain is able to process auditory information more efficiently and produce better behavioral outcomes when visual speech cues are available.

According to the theory of an integrated perceptual-cognitive system proposed by Schneider and Pichora-Fuller (2000), the observed perceptual benefit of AV speech should lead to more resources being available for higher-order cognitive processes, such as encoding of information in memory, and thus improved behavioral performance. This has been confirmed by Pichora-Fuller (1996) who demonstrated that visual speech cues help to counteract the negative effect of noise on working memory (WM) performance. We have previously examined the effect of AV speech on WM using an *n*-back task while also measuring ERP responses (Frtusova et al., 2013). The *n*-back task has been found to be sensitive to age-related changes (e.g., Verhaeghen and Basak, 2005; Van Gerven et al., 2007, 2008; Vaughan et al., 2008; Vermeij et al., 2012) and it has been examined by previous ERP research. It has been found that P3 amplitude decreases with increased WM load (i.e., higher n-back condition; Segalowitz et al., 2001; Watter et al., 2001), while P3 latency seems independent of *n*-back manipulation (Watter et al., 2001; Gaspar et al., 2011). These results were interpreted as suggesting that P3 amplitude reflects demands related to updating of WM, with greater demands resulting in a lower P3 amplitude, while P3 latency reflects processing related to the comparison of the current stimulus with the one presented *n*-trials before (Watter et al., 2001).

During the *n*-back task used in our previous experiment (Frtusova et al., 2013) with normal-hearing younger and older adults, spoken digits were presented in either the visual-only (V-only), A-only, or AV modality. The results showed that participants were faster across all memory loads, and more accurate in the most demanding WM conditions (2- and 3-back) when stimuli were presented in the AV modality compared to in the A-only and the V-only modality. Furthermore, the AV modality was associated with facilitated perceptual processing as evidenced by an earlier-peaking auditory N1 component in both age groups, and a smaller auditory N1 amplitude in older adults in the AV condition compared to the A-only condition.

The aforementioned findings come mostly from studies of younger and older adults with normal hearing. There is evidence to suggest that individuals with hearing impairment also benefit from having speech presented in the AV modality in terms of improved speech recognition in noisy environment (Grant et al., 1998; Tye-Murray et al., 2007; Bernstein and Grant, 2009). Furthermore, Grant et al. (2007) found that, despite a lower performance in an A-only condition during a syllable recognition task, participants with hearing impairment performed similarly to normal-hearing individuals in an AV condition. Thus, there is an indication that visual speech cues can help older adults with hearing impairment to counteract the hearing difficulty experienced during A-only conditions.

There is a scarcity of ERP research examining AV speech perception in the hearing impaired population. In one study, Musacchia et al. (2009) measured auditory ERPs in a group of older adults with normal hearing and those with mild to moderate hearing loss during A-only, V-only, and AV speech perception. Participants were asked to watch and/or listen to a repeated presentation of a “bi” syllable. The results showed that the AV modality did not result in the same level of modulation of ERP components for the hearing impaired group as it did for the normal-hearing controls. Musacchia et al. (2009) interpreted these results as an indication that AV integration abilities are diminished in individuals with hearing impairment. Thus, the results of this study seem contradictory to the observed AV speech benefit reported in behavioral studies and more ERP studies are needed to clarify this issue.

Importantly, there is preliminary behavioral evidence that older adults with hearing impairment may derive a WM benefit from AV speech. Brault et al. (2010) asked older adults with normal hearing and those with mild/moderate hearing loss to recall the last three words from word lists of unpredictable lengths. The word lists were presented in either the AV or the A-only modality. The results showed that when the stimuli were not perceptually degraded by white noise, older adults with hearing impairment and good lip-reading ability benefited from AV speech in comparison to A-only speech. On the other hand, when the stimuli were presented in background noise, the AV speech benefit in comparison to the A-only condition was evident independent of hearing impairment status or lip-reading proficiency. However, Brault et al. (2010) thought that these improvements were more related to perception rather than WM.

Overall, AV speech seems to improve speech recognition in individuals with hearing impairment, and there is preliminary evidence that it may also lead to better WM performance. However, more studies that include a combination of behavioral and electrophysiological measures are needed to provide information about the AV interaction effect in individuals with hearing impairment. ERP methodology, in particular, can help to clarify the timing and nature of the AV interaction in this population in comparison to normal-hearing controls. In addition, this methodology can also help to clarify to what extent the behavioral WM improvements are in fact related to perceptual facilitation of auditory processing during AV speech.

The present study examined the effect of AV speech on WM in older adults with hearing impairment in comparison to age- and education-matched controls. WM was tested using an *n*-back task with 0-, 1-, and 2-back conditions, and with A-only, V-only, and AV stimuli. During the task, ERP responses were collected together with behavioral accuracy and reaction time (RT) measures.

Similar to our previous work (Frtusova et al., 2013), it was expected that participants would have higher accuracy and faster RT in the AV condition compared to the A-only and V-only conditions. In addition, both perceptual and WM facilitation was expected to be evident on ERP measures in the AV condition compared to the A-only condition. More specifically, participants were expected to have earlier-peaking and smaller amplitude auditory P1 and N1 components during the AV condition compared to the A-only condition, indicating perceptual facilitation. Furthermore, they were expected to have an earlier-peaking and greater amplitude P3 component during the AV condition compared to the A-only condition, indicating WM facilitation. Note that perceptual facilitation is indicated by *smaller* P1 and N1 amplitudes as this suggests that fewer resources are required for auditory processing whereas WM facilitation is indicated by *greater* P3 amplitude as this suggests that more resources are available for WM processing.

Based on the hypothesis that strenuous perceptual processing caused by hearing impairment affords fewer available cognitive resources for higher-order functions, and the expectation that this effect can be counteracted by AV speech cues, we predicted a greater AV benefit for the hearing impaired population.

Furthermore, we examined whether a direct relationship between perceptual facilitation and improvement on WM could be found. A greater facilitation of N1 amplitude, indicating more efficient perceptual processing, was expected to be associated with higher accuracy. Additionally, a greater facilitation of N1 latency, indicating faster perceptual processing, was expected to be associated with faster RT.

## Materials and methods

### Participants

The sample in this study consisted of 16 older adults with poorer-hearing (PH) and 16 older adults with better-hearing (BH). Participants were recruited through the community, mostly an existing laboratory database, or through local advertisements and word of mouth by previous participants. Two PH participants were recruited through the Deaf and Hard-of-Hearing Program at the MAB-Mackay Rehabilitation Centre in Montreal and several were recruited through the Communicaid for Hearing Impaired Persons organization in Montreal. The data from 10 participants in the BH group came from a previous study (Frtusova et al., 2013) that used a nearly identical procedure (with the exception of eliminating the 3-back condition in this study). The analyses of behavioral data from the new participants compared to those from the previous study did not show any significant group differences (*M*_*diff*_ = 1.45, *p* = 0.39 for accuracy and *M*_*diff*_ = 21.22, *p* = 0.72 for RT). Thus, we chose to include all participants to increase statistical power.

All participants in this study were reasonably healthy, with no self-reported history of disease significantly affecting cognitive ability (e.g., stroke, dementia, Parkinson's disease, or epilepsy). All were completely fluent in English and were right handed (one participant in the PH group reported mixed handedness). Potential participants for the PH group were included if they reported hearing difficulty and either wore a hearing aid or were eligible for hearing aids according to their self-report. In this way we tried to limit our sample to participants with sensorineural hearing loss.

All participants completed a hearing screening that measured hearing thresholds for 250, 500, 1000, 2000, 4000 Hz (Welch Allyn, AM 232 Manual Audiometer). From these, we computed pure tone average (PTA) values for each ear by averaging across the thresholds obtained for 500, 1000, and 2000 Hz. Control participants had to have a PTA equal to or below 25 dB (Katz, 1985). The individuals in the PH group had to have sufficient hearing to be able to correctly identify the stimuli in the A-only condition without a hearing aid. All participants completed a vision screening that measured contrast sensitivity using the Mars Contrast Sensitivity Test (by MARS Perceptrix; Arditi, 2005). In this test, participants were asked to read a series of large print letters that were degraded in terms of background contrast. Contrast sensitivity, measured as logMAR scores, was obtained for each eye separately as well as binocularly. Lastly, cognitive screening was completed using the Montreal Cognitive Assessment (MoCA; Nasreddine et al., 2005). The groups were matched on age, education, gender, vision, and general cognitive skills. The demographic characteristics of the two samples are presented in Table 1. The protocol was approved by the University Human Research Ethics Committee (UHREC) of Concordia University as well as by the Review Ethics Board of CRIR Institutions.

**Table 1:** **Demographic characteristics**.

	**BH group (*n* = 16)**	**PH group (*n* = 16)**	
Males/Females	2/14	1/15	
Age (Years)	76.6 (4.93)	76.4 (9.57)	*p* > 0.05
Education (Years)	14.1 (2.53)	14.5 (3.45)	*p* > 0.05
MoCA^a^	27.5 (1.41)	26.3 (2.52)	*p* > 0.05
Binocular Vision (logMAR^b^)	1.7 (0.06)	1.7 (0.07)	*p* > 0.05
PTA^c^ Right Ear (dB)	15.6 (6.23)	47.8 (10.23)	*p* < 0.001
PTA^c^ Left Ear (dB)	15.4 (5.59)	49.4 (11.75)	*p* < 0.001

a *Montreal Cognitive Assessment (MoCA; Nasreddine et al., 2005)*.

b *Contrast sensitivity scores on Mars Contrast Sensitivity Test (Arditi, 2005)*.

c *The pure tone average (PTA) represents the average of hearing thresholds for 500, 1000, and 2000 Hz*.

### Stimuli

The stimuli consisted of short videos of a female speaking the digits 1, 2, 3, 4, 5, 6, 8, 9, and 10 with a neutral facial expression. The digit 7 was omitted because it is bi-syllabic and thus more easily distinguishable from the other digits. The stimuli were recorded in a recording studio at the Department of Journalism, Concordia University, and subsequently edited using Adobe Premier (Video codec, Windows Media Video 9; frame size, 500 px 388 px; frame rate, 29.97 fps; Audio codec, Windows Media Audio; sample rate and size, 44,100 Hz 16-bit). The videos showed the full face and shoulders of the speaker against a green background. The videos were edited such that the first obvious lip movement occurred nine frames after the onset of the video and the last lip movement happened approximately nine frames before the video ended. Imperceptible triggers were inserted at the time of the first lip movement (i.e., visual trigger) and at the onset of the sound (i.e., auditory trigger), in order to signal these events to the recording electroencephalogram amplifier which was important for subsequent ERP analyses (as described later). The lag between the onset of the video and the onset of the sound was approximately 395.3 ms (*SD* = 103.24). The average length of the video was 2010 ms (*SD* = 160 ms), with an inter-trial interval of 2400 ms. The sound was presented binaurally using insert earphones (EARLINK tube ear inserts; Neuroscan, El Paso, Texas).

The AV stimuli included both video and audio channels, meaning that the participants could both see and hear the speaker. For the A-only stimuli, the video channel was deleted and only a white fixation point was presented on a black background to maintain eye fixation. For the V-only stimuli, the auditory channel was deleted and the participants needed to identify the digits based on the visual speech cues. Overall, the stimuli in the three modalities were identical with the exception of the presence of either both of the modalities or only one of the modalities. The stimuli were presented on a black screen 15-in. CRT monitor, using Inquisit (version 2.0; Millisecond Software, 2008). Participants were seated in a comfortable chair approximately 60 cm from the screen.

### Procedure

Participants completed the *n*-back task in three modalities: V-only (where they could see the speaker presenting the digits but could not hear her voice); A-only (where they could hear the speaker presenting digits but could not see her face); and AV (where they could both hear and see the speaker presenting digits). There were three different levels of task difficulty ranging from 0-back to 2-back load in a blocked design. In the 0-back condition, participants had to decide whether the currently presented digit matched a target digit assigned at the beginning of the block. In the 1-back condition, participants had to decide whether the currently presented digit matched the one presented one trial before, and in the 2-back condition, participants had to decide whether the currently presented digit matched the one presented two trials before.

The sequences of digits were semi-random, each containing 40 “Match” trials and 60 “Non-Match” trials. In Match trials, the currently presented digit matched the one assigned at the beginning of the block (0-back) or the one presented one or two trials before (1- and 2-back, respectively). Participants completed the 0-back condition in each modality, followed by the 1-back condition in each modality and finished with the 2-back condition in each modality. The order of the modality presentations was varied across participants. Participants were presented with different sequences of digits in different modalities, but modality-sequence combinations were also varied across participants.

Participants practiced speech-reading and responding with the computer mouse before the experiment began. To practice speech-reading, participants had to identify the digits used in the experiment based on only seeing the speaker to utter these digits (similar to the V-only condition). Digits were first presented in numerical and then random order. This procedure was repeated if the participant made mistakes in the random practice condition. In general, participants had to identify all the digits correctly in the practice session before proceeding with the experiment. To practice responding with the computer mouse, participants were asked to hold the mouse in both of their hands and press the left or right button using their thumbs to indicate Match or Non-Match responses. The assignment of Match response to the left or right button was counterbalanced across participants. For all conditions, participants were instructed to respond as fast and as accurately as they could. To practice responding, they completed 10 trials that were identical to the AV 0-back condition. After this, the experimental tasks began. In order to ensure that each participant understood the task, they completed 10 practice trials before each new *n*-back block (i.e., before beginning the 0-back, 1-back, and 2-back tasks). During these trials, feedback was provided by presenting a short low-frequency beep whenever participants made a mistake. The practice blocks were repeated if participants made more than a few mistakes or it appeared that they did not understand the task. For many participants, this was mostly necessary in the 2-back condition. Lastly, in order to give participants a chance to adjust to each new condition, five “Warm-Up” trials were included at the beginning of each sequence. These trials were not counted in the analyses.

Two behavioral measures were collected: the accuracy, defined as the percentage of correct Match responses, and RT, defined as the amount of time between the onset of the auditory trigger and the participant's button response for correct Match trials. Trials were excluded if the response occurred less than 200 ms after the first cue about the identity of the digit (i.e., the onset of the lip movement in the V-only and AV conditions or the onset of the sound in the A-only condition). This was done because such early responses were unlikely to represent a valid response.

### Electroencephalography data acquisition and processing

The electroencephalography (EEG) data were collected during the task using a Biosemi ActiveTwo system with 72 channels. Sixty-four electrodes were arranged on the head according to the extended International 10–20 system (Jasper, 1958). Electro-occulograms (EOG) were used to monitor eye movements: one electrode was placed above and one below the left eye to monitor vertical eye movements and one was placed beside the outer canthi of each eye to monitor horizontal eye movements. The sampling rate during the recording was 2048 Hz but the files were down-sampled offline to 512 Hz.

After down-sampling, the recorded data were converted to Neuroscan continuous data format using Polygraphic Recording Data Exchange (PolyRex; Kayser, 2003). The data were re-referenced to a linked left and right ear lobe reference and subsequently processed using Scan software (version 4.5; Compumedics Neuroscan, 2009). Vertical ocular artifacts were corrected using a spatial filtering technique (Method 1; NeuroScan Edit 4.5 manual, 2009). Next, the frequencies outside the range of 1–45 Hz were filtered using a bandpass filter. Continuous recordings were divided into separate epochs going from −100 to 1000 ms around the onset of auditory stimuli (i.e., auditory triggers) and baseline corrected based on the 100 ms prestimulus period (i.e., −100 to 0 ms before the auditory trigger). Epochs with excessive artifacts (i.e., activity larger than ±75 μV in the active electrodes at and around the midline or EOG activity exceeding ±60 μV) were excluded by the software program. The accepted epochs were subsequently inspected manually by the examiner to ensure that there was no excessive noise in the epochs that were to be used in the analyses. The mean number of accepted trials was 31.5 out of 40 for the Match condition (*SD* = 5.64). The epochs were then sorted by the software based on the condition, and individual averages (i.e., average waveforms for each individual) for each condition were computed. In order to examine the AV interaction, the waveforms for A-only and V-only were added to create A+V waveforms (Klucharev et al., 2003; van Wassenhove et al., 2005; Pilling, 2009; Winneke and Phillips, 2011; Frtusova et al., 2013).

In this study, we were interested in three ERP components, namely the P1, N1, and P3. These components were first detected by a semiautomatic procedure in Scan software (NeuroScan Edit 4.5 manual). For this purpose, the P1 was defined as the highest positive point occurring between 20 and 110 ms after the onset of the stimulus; the N1 was defined as the lowest negative point occurring between 60 and 170 ms after the onset of the stimulus; and the P3 was defined as the most positive point occurring between 300 and 700 ms after the onset of the stimulus. Subsequently, the detected peaks were inspected and manually adjusted, when necessary, by a trained examiner who was blinded to the modality and group factors.

## Results

The data were analyzed by repeated measures ANOVAs using SPSS (version 22; IBM). Predicted interaction effects were decomposed with simple effects analyses. The reported results are significant at α ≤ 0.05 unless otherwise specified. For the main analyses, the Greenhouse-Geisser non-sphericity correction was used for interpreting results for within-subject factors with more than two levels. Based on the convention suggested by Jennings (1987), Greenhouse-Geisser epsilon (ε) values and uncorrected degrees of freedom are reported together with adjusted *p*-values and mean square error (*MSE*) values. Participants had to reach an accuracy of at least 60% during a particular condition in order to be included in the analyses; otherwise the value for that condition was replaced by the group mean. This criterion was imposed in order to ensure that participants were sufficiently engaged in the task so that the observed values indicated a valid representation of task-related performance. Eight values (out of 144) needed to be replaced in the BH group and nine values (out of 141) needed to be replaced in the PH group. In addition, one participant from the PH group discontinued the 2-back condition because she found it too difficult and thus the missing values were replaced by group means.

### Behavioral results

Behavioral data were analyzed by repeated measures ANOVAs with modality (V-only, A-only, AV) and *n*-back load (0-, 1-, and 2-back) entered as within-subject variables and group (BH and PH) entered as a between-subject variable.

#### Accuracy

**Figure 1:**
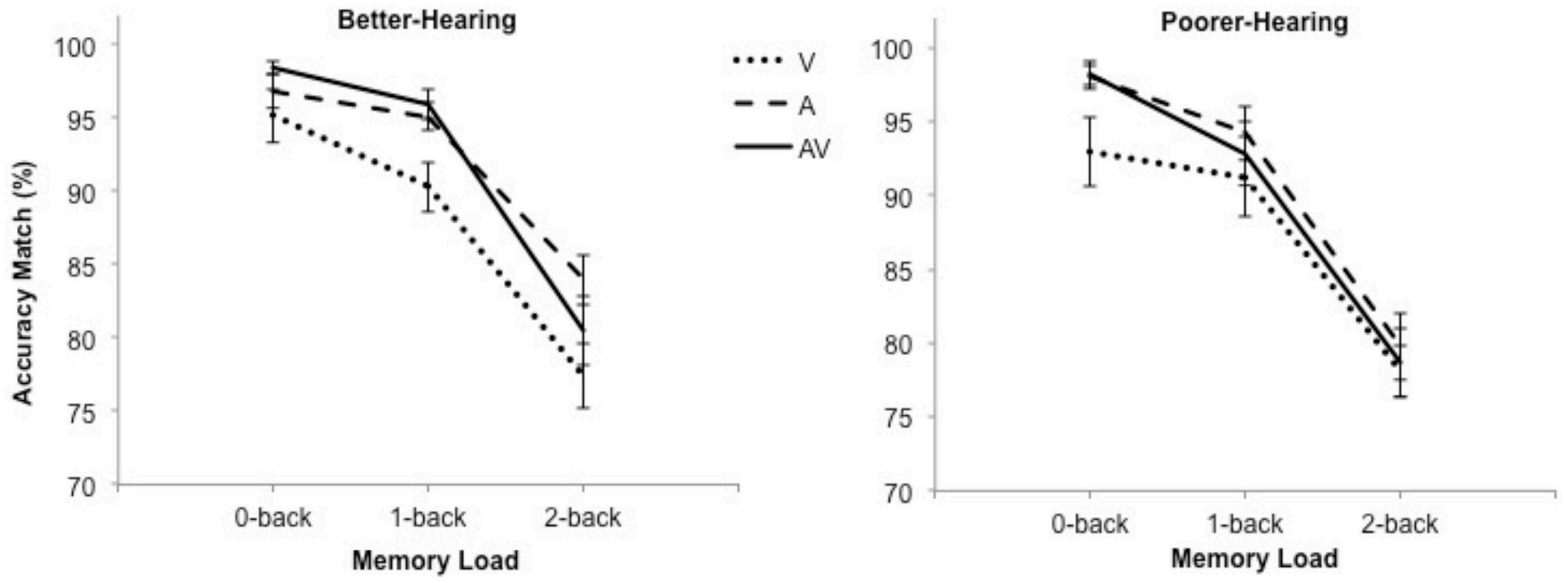
**The mean percentage of correct responses and standard error bars for better-hearing participants (left panel) and participants with poorer hearing (right panel)**.

The accuracy data are shown in Figure 1. The analysis revealed a significant main effect of modality [*F*_(2, 60)_ = 9.7; *MSE* = 48.22; *p* < 0.001; ε = 0.86; $\eta_{p}^{2}=0.25$], indicating that participants were more accurate in the A-only and AV conditions compared to the V-only condition but performance in the A-only and the AV condition did not differ. There was also a main effect of load [*F*_(2, 60)_ = 162.2; *MSE* = 53.23; *p* < 0.001; ε = 0.89; $\eta_{p}^{2}=0.84$], showing that accuracy decreased as *n*-back load increased. Neither the main effect of group [*F*_(1, 30)_ = 0.6; *MSE* = 127.95; *p* = 0.43; $\eta_{p}^{2}=0.02$] nor the Modality × Group interaction [*F*_(2, 60)_ = 0.4; *MSE* = 48.22; *p* = 0.66; ε = 0.86; $\eta_{p}^{2}=0.01$] were significant.

#### Reaction time

**Figure 2:**
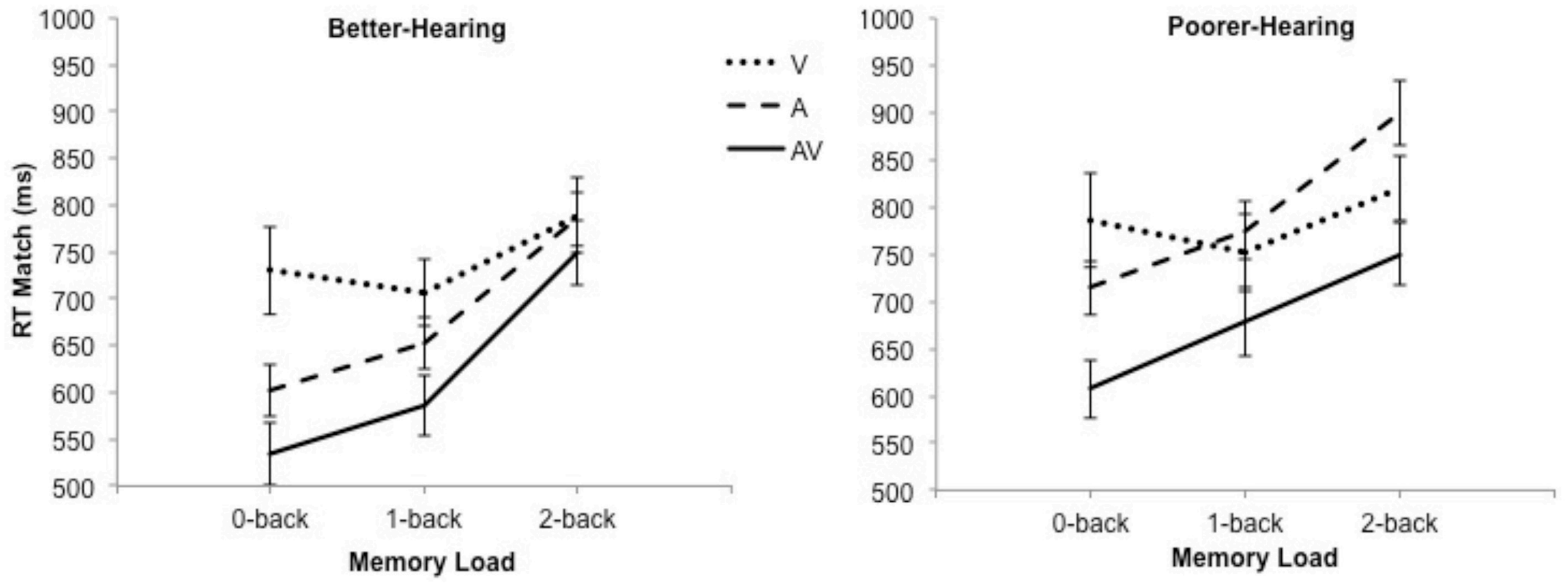
**The mean reaction time and standard error bars for better-hearing participants (left panel) and participants with poorer hearing (right panel)**. Note the faster responses during the AV condition in comparison to the A-only condition in both groups.

The RT data are shown in Figure 2. The analysis revealed a significant main effect of modality [*F*_(2, 60)_ = 42.6; *MSE* = 9946.64; *p* < 0.001; ε = 0.80; $\eta_{p}^{2}=0.59$], load [*F*_(2, 60)_ = 29.7; *MSE* = 18372.55; *p* < 0.001; ε = 0.91; $\eta_{p}^{2}$ = 0.50], as well as a significant Modality × Load interaction [*F*_(4, 120)_ = 7.8; *MSE* = 9575.33; *p* < 0.001; ε = 0.66; $\eta_{p}^{2}$ = 0.21]. Pairwise comparisons showed that participants were faster during the AV condition compared to the V-only and A-only conditions at all *n*-back loads, but they were faster in the A-only condition compared to the V-only condition only during the 0-back condition.

Furthermore, there was a statistical trend for the effect of group [*F*_(1, 30)_ = 3.6; *MSE* = 105309.68; *p* = *0.0*7; $\eta_{p}^{2}$ = 0.11], indicating that the BH group was faster than the PH group. This effect was qualified by a Modality × Group interaction [*F*_(2, 60)_ = 4.6; *MSE* = 9946.64; *p* = 0.02; ε = 0.80; $\eta_{p}^{2}$ = 0.13], which showed that the PH group performed similarly to the BH group in the V-only [*F*_(1, 30)_ = 0.8; *p* = 0.37; $\eta_{p}^{2}$ = 0.03] and the AV [*F*_(1, 30)_ = 2.0; *p* = 0.17; $\eta_{p}^{2}=0.06$] conditions but were significant slower in the A-only condition [*F*_(1, 30)_ = 12.6; *p* = 0.001; $\eta_{p}^{2}=0.30$].

### Electrophysiological results: perceptual processing

For the electrophysiological results, the V-only condition was not included in the analyses because our analyses focused on the auditory evoked potentials. More specifically, we were interested in the comparison of auditory processing with and without the presence of visual speech cues. N1 amplitude was defined as an absolute voltage difference between the trough of the P1 and the peak of the N1, thus we refer to this component complex as P1-N1 when describing the amplitude data. In order to explore the possibility of multisensory effects occurring before the N1 component, we also analyzed the data from the P1 component separately. P1 amplitude was measured relative to the 0 μV baseline. The P1 and N1 latencies were measured at the components' peaks relative to the onset of the auditory trigger. The data from the CZ electrode were used for the analyses as these components reach their maximum in mid-central electrodes (Näätänen and Picton, 1987) and no hemispheric differences were identified in a previous work in our laboratory (Winneke and Phillips, 2011).

To explore multisensory processing, the AV and the A-only conditions were compared to the A+V measure. This waveform was obtained by the summation of electrophysiological activity in the A-only and the V-only conditions locked to the onset of the auditory stimuli. For this purpose, we embedded imperceptible triggers into the V-only files at the time points where the onset of the sound would have occurred, if it had been presented (i.e., at the identical time point as in the A-only and the AV stimuli). This way we were able to assess whether the AV condition represented a multisensory interaction or merely the simultaneous processing of two independent modality channels (A-only and V-only). Planned comparisons consisted of the contrast of A-only vs. AV waveforms and A-only vs. A+V waveforms. The values for the P1 and P1-N1 amplitudes and for the P1 and N1 latencies were analyzed by repeated measures ANOVAs with modality (AV, A-only, A+V) and *n*-back load (0-, 1-, and 2-back) conditions entered as within-subject variables and group (BH and PH) entered as a between- subject variable.

#### P1-N1 amplitude

**Figure 3:**
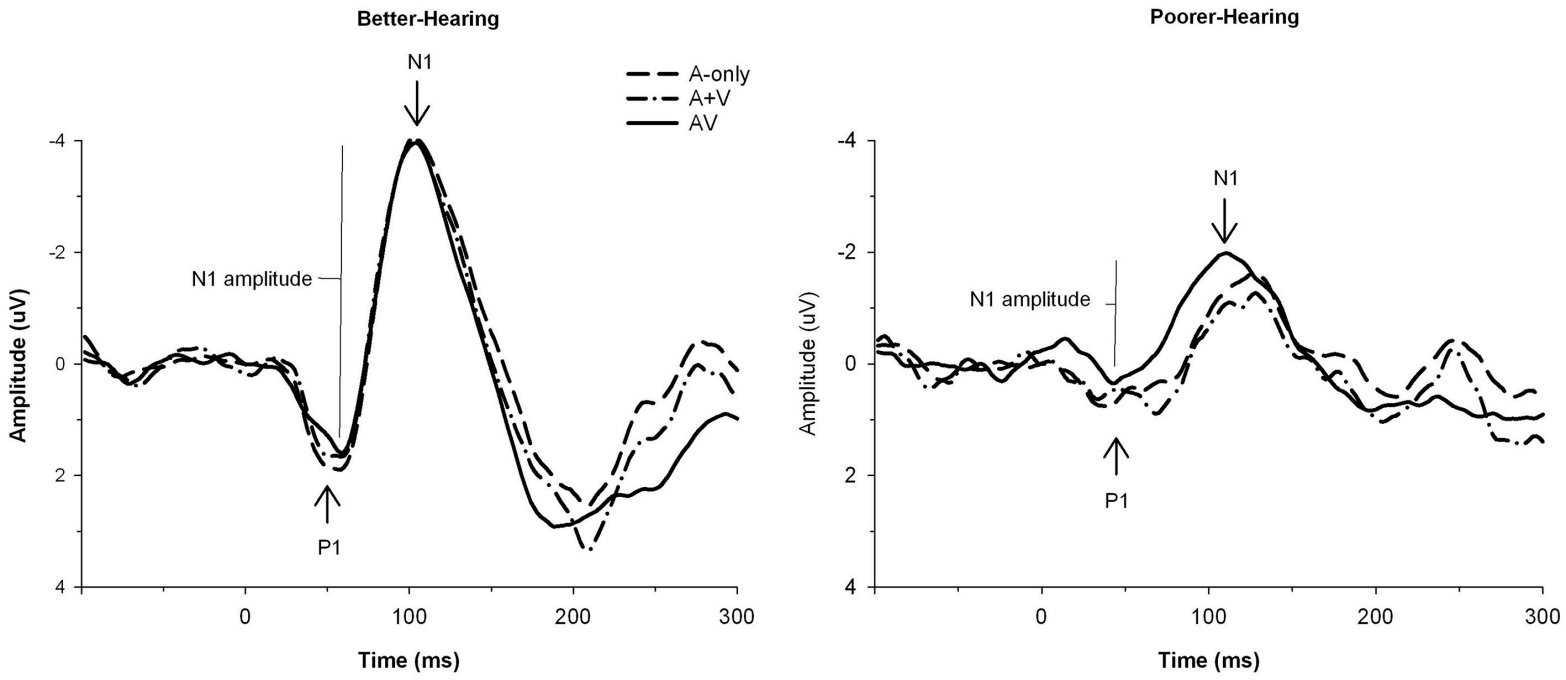
**The grand average waveforms of auditory event-related potentials at the CZ electrode, illustrating the amplitudes and latencies of the P1 and N1 components for better-hearing older adults (left panel) and older adults with poorer hearing (right panel)**. The data are collapsed across different *n*-back conditions. Note the smaller amplitude of P1 and N1, and earlier P1 in the AV in comparison to the A-only condition for participants with poorer hearing.

The grand averages illustrating different modalities for the P1-N1 wave are presented in Figure 3. The mean values and standard deviations are also presented in Table 2. The ANOVA showed a main effect of modality [*F*_(2, 60)_ = 12.5; *MSE* = 4.74; *p* < 0.001; ε = 0.74; $\eta_{p}^{2}$ = 0.29], such that the amplitude of the P1-N1 was smaller in the AV condition compared to both the A-only condition and the A+V measure, and smaller in the A-only condition compared to the A+V measure. Thus, the data provided evidence for a multisensory interaction in the AV condition. There was also a main effect of group [*F*_(1, 30)_ = 10.5; *MSE* = 38.88; *p* = *0.0*03; $\eta_{p}^{2}$ = 0.26] with the PH group having a smaller P1-N1 amplitude than the BH group.

**Table 2:** **The mean amplitudes (μV) and standard deviations (in parenthesis) of the P1-N1 component for better-hearing (BH) participants and participants with poorer hearing (PH) at the CZ electrode**.

**Load**	**Modality**		
	**A-only**	**A+V**	**AV**
**BH GROUP**			
0-back	8.3 (0.71)	8.8 (0.74)	6.9 (0.69)
1-back	7.1 (0.61)	7.3 (0.76)	6.7 (0.61)
2-back	7.9 (0.59)	8.0 (0.66)	7.7 (0.74)
**PH GROUP**			
0-back	5.2 (0.71)	5.9 (0.74)	4.3 (0.69)
1-back	5.5 (0.61)	6.6 (0.76)	4.5 (0.61)
2-back	5.3 (0.59)	5.8 (0.66)	4.2 (0.74)

In order to test our main hypothesis, the planned simple effects, followed by pairwise comparisons, indicated that there was a significant decrease in P1-N1 amplitude in the AV condition compared to the A-only condition and the A+V measure, and in the A-only condition compared to the A+V measure for the PH group [*F*_(2, 29)_ = 7.4; *p* = 0.003; $\eta_{p}^{2}=0.34$]. However, while a similar pattern of results was suggested in the BH group, the mean differences did not reach the level of significance [*F*_(2, 29)_ = 1.8; *p* = 0.19; $\eta_{p}^{2}$ = 0.11; see Table 2].

#### P1 amplitude

The grand averages illustrating different modalities for the P1 wave are presented in Figure 3. The mean values and standard deviations are also presented in Table 3. The ANOVA showed a main effect of modality [*F*_(2, 60)_ = 10.2; *MSE* = 4.62; *p* = 0.001; ε = 0.76; $\eta_{p}^{2}$ = 0.25]; the amplitude of P1 was smaller in the AV condition compared to the A-only condition and the A+V measure, while the A-only condition and the A+V measure did not significantly differ. These results indicate that the multisensory interaction effect is evident early in the information processing stream and modulation observed in the AV condition compared to the A-only condition cannot be explained by simultaneous but independent processing of visual and auditory speech information.

**Table 3:** **The mean amplitudes (μV) and standard deviations (in parenthesis) of the P1 component for better-hearing (BH) participants and participants with poorer hearing (PH) at the CZ electrode**.

**Load**	**Modality**		
	**A-only**	**A+V**	**AV**
**BH GROUP**			
0-back	3.3 (0.37)	3.4 (0.53)	2.1 (0.56)
1-back	1.9 (0.47)	2.1 (0.60)	2.0 (0.57)
2-back	3.0 (0.33)	2.9 (0.55)	3.2 (0.42)
**PH GROUP**			
0-back	2.4 (0.37)	2.8 (0.53)	1.1 (0.56)
1-back	1.8 (0.47)	2.8 (0.60)	0.5 (0.57)
2-back	2.2 (0.33)	2.7 (0.55)	0.8 (0.42)

There was also a main effect of group [*F*_(1, 30)_ = 4.4; *MSE* = 9.35; *p* = *0.0*4; $\eta_{p}^{2}$ = 0.13], with the PH group having a smaller P1 amplitude than the BH group. Furthermore, there was a significant Modality × Group interaction [*F*_(2, 60)_ = 4.2; *MSE* = 4.62; *p* = 0.03; ε = 0.76; $\eta_{p}^{2}$ = 0.12], indicating that for the PH group [*F*_(2, 29)_ = 9.0; *p* = *0.0*01; $\eta_{p}^{2}$ = 0.38], P1 amplitude was smaller in the AV condition compared to the A-only condition and the A+V measure, and there was a statistical trend (*p* = 0.06) for the P1 to be smaller in the A-only condition compared to the A+V measure. However, no modality effect was indicated in the BH group, [*F*_(2, 29)_ = 0.5; *p* = 0.61; $\eta_{p}^{2}$ = 0.03; see Table 3]. The simple effects conducted on the interaction also revealed that the PH group had a smaller P1 amplitude in the AV condition compared to the BH group [*F*_(1, 30)_ = 11.9; *p* = 0.002; $\eta_{p}^{2}$ = 0.28], while the two groups had similar P1 amplitudes in the A-only condition [*F*_(1, 30)_ = 2.1; *p* = 0.16; $\eta_{p}^{2}$ = 0.07] and the A+V measure [*F*_(1, 30)_ = 0.02; *p* = 0.90; $\eta_{p}^{2}$ = 0.00]. Lastly, there was a main effect of load [*F*_(2, 60)_ = 3.5; *MSE* = 4.86; *p* = 0.05; ε = 0.84; $\eta_{p}^{2}$ = 0.10] with P1 amplitude being smaller in the 1-back than the 0-back condition. No other differences were evident across different WM loads.

#### P1 latency

The grand averages illustrating different modalities for the P1 wave are presented in Figure 3. The mean values and standard deviations are also presented in Table 4. The data showed the main effect of modality [*F*_(2, 60)_ = 6.0; *MSE* = 373.14; *p* = 0.01; ε = 0.86; $\eta_{p}^{2}$ = 0.17]; the P1 peaked earlier in the AV condition compared to the A-only condition and the A+V measure, while the A-only condition and the A+V measure did not significantly differ. The main effect of group was not significant [*F*_(1, 30)_ = 2.9; *MSE* = 854.68; *p* = *0.1*0; $\eta_{p}^{2}$ = 0.09] but there was a statistical trend toward a Modality × Group interaction [*F*_(2, 60)_ = 3.2; *MSE* = 373.14; *p* = 0.06; ε = 0.86; $\eta_{p}^{2}$ = 0.10], indicating that the P1 peaked earlier in the AV condition compared to the A-only condition and the A+V measure for the PH group [*F*_(2, 29)_ = 7.1; *p* = 0.003; $\eta_{p}^{2}$ = 0.33] but the differences in the BH group did not reach statistical significance [*F*_(2, 29)_ = 0.3; *p* = *0.78*; $\eta_{p}^{2}=0.02$].

**Table 4:** **The mean latencies (ms) and standard deviations (in parenthesis) of the P1 component for better-hearing (BH) participants and participants with poorer hearing (PH) at the CZ electrode**.

**Load**	**Modality**		
	**A-only**	**A+V**	**AV**
**BH GROUP**			
0-back	47.1 (4.89)	45.0 (4.36)	49.2 (3.74)
1-back	50.6 (4.92)	50.4 (6.06)	48.6 (4.57)
2-back	50.2 (3.31)	53.4 (4.61)	44.1 (4.16)
**PH GROUP**			
0-back	59.5 (4.89)	66.5 (4.36)	52.6 (3.74)
1-back	54.7 (4.92)	60.6 (6.06)	40.3 (4.57)
2-back	51.9 (3.31)	58.6 (4.61)	46.6 (4.16)

#### N1 latency

The grand averages illustrating different modalities for the N1 wave are presented in Figure 3. The mean values and standard deviations are presented in Table 5. The main effect of modality did not reach statistical significance [*F*_(2, 60)_ = 3.0; *MSE* = 600.15; *p* = 0.09; ε = 0.61; $\eta_{p}^{2}$ = 0.09]. There was a statistical trend toward the main effect of group [*F*_(1, 30)_ = 3.8; *MSE* = 1311.89; *p* = 0.06; $\eta_{p}^{2}$ = 0.11], with the N1 peaking later in the PH group than the BH group. There was a main effect of load [*F*_(2, 60)_ = 5.6; *MSE* = 482.81; *p* = 0.01; ε = 0.95; $\eta_{p}^{2}$ = 0.16], which was qualified by a Load × Group interaction [*F*_(2, 60)_ = 4.3; *MSE* = 482.81; *p* = 0.02; ε = 0.95; $\eta_{p}^{2}$ = 0.13] and further by a Modality × Load × Group interaction [*F*_(4, 120)_ = 2.8; *MSE* = 376.14; *p* = 0.05; ε = 0.69; $\eta_{p}^{2}$ = 0.09]. The simple effects and pairwise comparisons indicated that there were no statistical differences in the BH group (all *Fs* < 1.9; all *p*s > 0.16). For the PH group, no differences across different modalities were observed in the 0-back [*F*_(2, 29)_ = 0.2; *p* = 0.81; $\eta_{p}^{2}=0.02$] condition, but the N1 peaked earlier in the AV condition compared to the A-only condition and the A+V measure during the 1-back load [*F*_(2, 29)_ = 6.5; *p* = 0.01; $\eta_{p}^{2}$ = 0.31], and earlier in the A-only condition compared to the A+V measure during the 2-back load [*F*_(2, 29)_ = 3.1; *p* = 0.06; $\eta_{p}^{2}=0.18$].

**Table 5:** **The mean latencies (ms) and standard deviations (in parenthesis) of the N1 component for better-hearing (BH) participants and participants with poorer hearing (PH) at the CZ electrode**.

**Load**	**Modality**		
	**A-only**	**A+V**	**AV**
**BH GROUP**			
0-back	101.2 (7.16)	102.8 (6.97)	99.8 (4.60)
1-back	104.5 (5.68)	103.1 (5.15)	102.4 (4.64)
2-back	102.2 (4.08)	100.3 (4.53)	94.5 (3.31)
**PH GROUP**			
0-back	118.5 (7.16)	121.7 (6.97)	119.9 (4.60)
1-back	115.0 (5.68)	115.0 (5.15)	90.9 (4.64)
2-back	97.7 (4.08)	105.4 (4.53)	101.9 (3.31)

### Electrophysiological results: working memory processing

P3 amplitude was measured relative to the 0 μV baseline and P3 latency was measured at the component's peak relative to the onset of the auditory trigger. The data from the PZ electrode were used for the analyses as this component reaches its maximum in mid-posterior sites (Watter et al., 2001; Frtusova et al., 2013). The P3 is considered to reflect WM processes (i.e., higher-order) rather than perceptual processing and thus for this condition we only compared the AV and A-only modalities. The values from the P3 components were analyzed by repeated measures ANOVAs with the modality (A-only and AV) and *n*-back load (0-, 1-, and 2-back) conditions entered as within-subject variables and group (BH and PH) entered as a between-subject variable.

#### P3 amplitude

**Figure 4:**
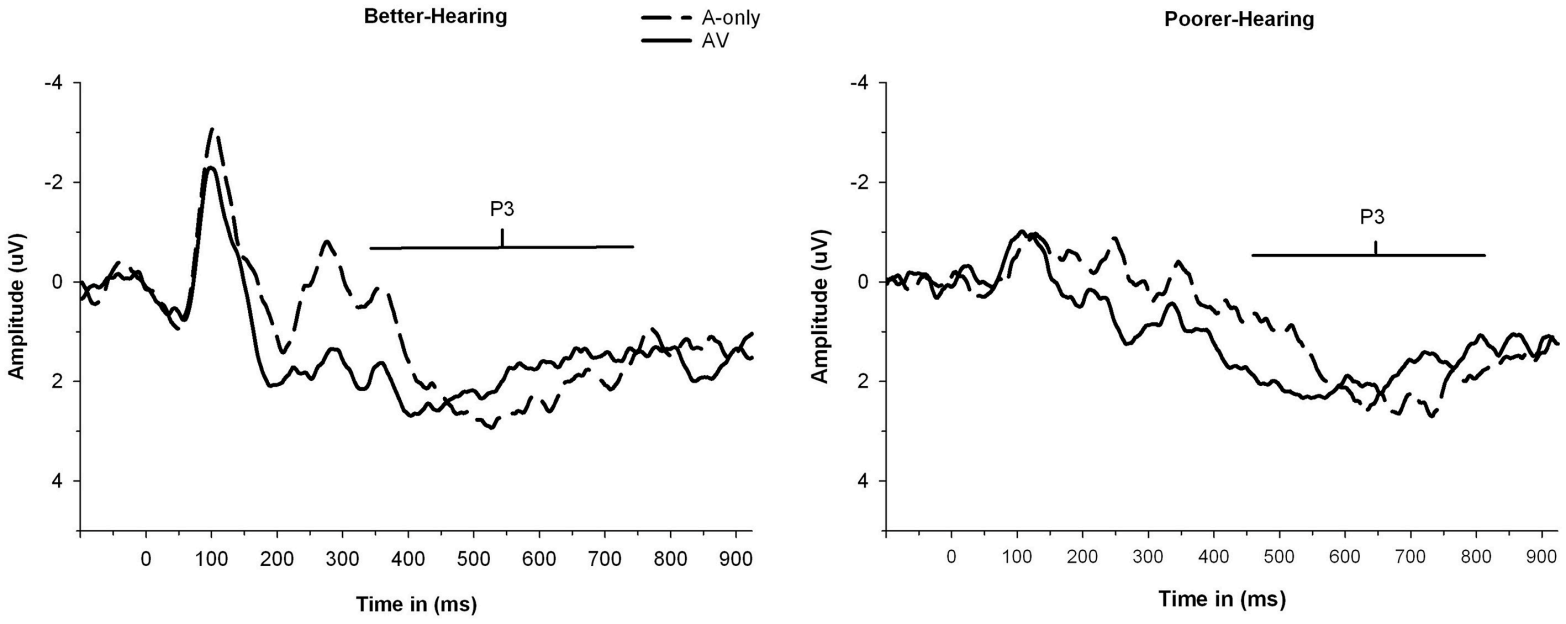
**The grand average waveforms of auditory event-related potentials at PZ electrode, illustrating the amplitudes and latencies of P3 component for better-hearing older adults (left panel) and older adults with poorer hearing (right panel)**. The data are collapsed across different *n*-back conditions. Note the smaller P3 amplitude in participants with poorer hearing for the A-only condition but similar P3 amplitudes in both groups for the AV condition. Also note the earlier peaking P3 in the AV in comparison to the A-only condition in both groups and later peaking P3 in both modalities for participants with poorer hearing.

The grand averages illustrating different modalities for the P3 wave are presented in Figure 4. The mean values and standard deviations are also presented in Table 6. The ANOVA showed that neither the main effect of modality [*F*_(1, 30)_ = 0.5; *MSE* = 2.69; *p* = 0.50; $\eta_{p}^{2}$ = 0.02] nor the main effect of group [*F*_(1, 30)_ = 3.0; *MSE* = 15.58; *p* = 0.10; $\eta_{p}^{2}$ = 0.09] was significant. However, there was a significant Modality × Group interaction [*F*_(1, 30)_ = 4.1; *MSE* = 2.69; *p* = 0.05; $\eta_{p}^{2}$ = 0.12]. The two groups had similar P3 amplitudes in the AV condition [*F*_(1, 30)_ = 0.6; *p* = 0.43; $\eta_{p}^{2}$ = 0.02] but the PH group had significantly smaller P3 amplitude in the A-only condition compared to the BH group [*F*_(1, 30)_ = 5.8; *p* = 0.02; $\eta_{p}^{2}$ = 0.16]. As expected, there was a main effect of load [*F*_(2, 60)_ = 11.3; *MSE* = 5.04; *p* < 0.001; ε = 0.79; $\eta_{p}^{2}$ = 0.27], with P3 amplitude being greater in the 0-back condition compared to the 1-back and 2-back conditions, while the 1-back and 2-back conditions did not significantly differ.

**Table 6:** **The mean amplitudes (μV) and standard deviations (in parenthesis) of the P3 component for better-hearing (BH) participants and participants with poorer hearing (PH) at the PZ electrode**.

**Load**	**Modality**	
	**A-only**	**AV**
**BH GROUP**		
0-back	6.4 (0.66)	5.8 (0.70)
1-back	4.7 (0.52)	4.7 (0.55)
2-back	4.5 (0.47)	4.2 (0.51)
**PH GROUP**		
0-back	4.4 (0.66)	5.6 (0.70)
1-back	3.7 (0.52)	3.9 (0.55)
2-back	3.2 (0.47)	3.8 (0.51)

#### P3 latency

The grand averages illustrating the different modalities for the P3 wave are presented in Figure 4. The mean values and standard deviations are presented in Table 7. The data showed a main effect of modality [*F*_(1, 30)_ = 11.3; *MSE* = 5319.22; *p* = 0.002; $\eta_{p}^{2}$ = 0.27], with the P3 peaking earlier in the AV condition compared to the A-only condition. There was also a main effect of group [*F*_(1, 30)_ = 14.2; *MSE* = 13022.67; *p* = 0.001; $\eta_{p}^{2}$ = 0.32], with the P3 peaking later in the PH group compared to the BH group. The interaction between Modality × Group was not significant [*F*_(1, 30)_ = 0.02; *MSE* = 5319.22; *p* = 0.88; $\eta_{p}^{2}$ = 0.00].

**Table 7:** **The mean latencies (ms) and standard deviations (in parenthesis) of the P3 component for better-hearing (BH) participants and participants with poorer hearing (PH) at the PZ electrode**.

**Load**	**Modality**	
	**A-only**	**AV**
**BH GROUP**		
0-back	473.9 (15.20)	423.9 (17.75)
1-back	487.2 (17.49)	476.0 (22.61)
2-back	507.0 (13.25)	456.8 (14.96)
**PH GROUP**		
0-back	557.8 (15.20)	509.4 (17.75)
1-back	536.3 (17.49)	512.1 (22.61)
2-back	555.1 (13.25)	526.3 (14.96)

### Correlation between facilitation of perceptual processing and improvement in working memory performance

We examined whether there is a relationship between the amount of perceptual facilitation (i.e., a decrease in the amplitude of the P1-N1 and the latency of the auditory N1) and the level of behavioral improvement on the WM task in the AV condition compared to the A-only condition. Firstly, we examined whether there is a positive relationship between the facilitation of the auditory P1-N1 amplitude (A-only–AV) and higher accuracy (AV–A-only). Secondly, we examined whether there is a positive relationship between facilitation of the auditory N1 latency (A-only–AV) and faster RT (A-only–AV). We reasoned that participants with greater perceptual facilitation should have greater behavioral improvement. The results are presented in Table 8 (note that positive correlations always reflect a relationship in the expected direction).

**Table 8:** **Zero-order correlations between the facilitation of P1-N1 amplitude and improvement in accuracy (on the left) and facilitation of N1 latency and improvement in reaction time (RT; on the right) during the AV condition in comparison to A-only condition**.

	**P1-N1 Amplitude**		**N1 Latency**	
	**BH accuracy**	**PH accuracy**	**BH RT**	**PH RT**
0-back	0.26	−0.09	0.13	0.02
1-back	0.16	−0.11	0.31	0.05
2-back	0.13	0.34	0.43^*^	0.10

* *significant at α ≤ 0.05 one-tailed*.

## Discussion

This study examined the effect of AV speech on WM in older adults with hearing impairment compared to better-hearing older adults. The results showed that both groups were faster in the AV condition compared to the unisensory conditions even though the accuracy was comparable between the AV and A-only conditions. Participants with hearing impairment were slower compared to controls during the A-only condition but the two groups performed similarly in the AV and the V-only conditions. These results suggest that group differences in the A-only condition are due to more demanding perceptual processing for the PH group rather than differences in WM, and that visual speech cues can help to counteract this more demanding auditory processing.

The electrophysiological results revealed facilitation of perceptual processing in the PH group, indicated by smaller and faster perceptual ERP responses during the AV condition compared to the A-only condition. Furthermore, the ERP data showed facilitation of WM processing, indicated by earlier P3 components in both groups. For P3 amplitude, the PH group had smaller P3 amplitude than the BH group in the A-only condition but no group differences were observed in the AV condition, supporting the suggestion that visual speech cues can help to counteract the negative effect of more demanding perceptual processing on WM.

### Auditory-visual speech interaction in older adults with hearing impairment

The results of the current study indicate that older adults with hearing impairment show a more robust multisensory interaction effect compared to older adults with age-normal hearing. More specifically, the amplitudes of the auditory P1 and the P1-N1 were significantly reduced in the AV condition compared to the A-only condition and the A+V measure for participants with hearing impairment but these effects did not reach statistical significance in participants with age-normal hearing. Similarly, there was a reduction in the auditory P1 latency during the AV condition, compared to the A-only condition and the A+V measure, evident in hearing impaired participants while in those with age-normal hearing these differences were not statistically significant. Lastly, for the auditory N1 latency, a reduction in the AV condition compared to the A-only condition and the A+V measure, was observed in the 1-back load for the hearing impaired group while no significant differences were seen in controls. Overall, our results suggest intact AV multisensory interaction in older adults with hearing impairment. These effects were observed early in the processing stream (i.e., the level P1 component), suggesting that the multisensory interaction is occurring as early as at the level of the primary auditory cortex (Liegeois-Chauvel et al., 1994).

These results stand in contrast to those by Musacchia et al. (2009) who found that older adults with hearing impairment may not be able to integrate auditory and visual speech information to the same extent as older adults with age-normal hearing. There are several methodological differences between the current study and that conducted by Musacchia et al. (2009) that may have contributed to the differences in the results. For example, Musacchia et al. (2009) assessed speech perception by repetition of the same syllable, participants were not actively involved in the task, which may have affected their attention to the stimuli, and lastly, they equalized the auditory input across the groups by adjusting the intensity level of the stimuli. Our results confirmed the observation of improved perceptual functioning during AV speech reported by behavioral studies examining speech recognition in older adults with hearing impairment (e.g., Grant et al., 1998; Tye-Murray et al., 2007; Bernstein and Grant, 2009).

### The effect of auditory-visual speech on working memory

The behavioral results showed faster RT during the AV condition compared to the unisensory conditions in both groups, suggesting facilitation of WM processing. Furthermore, while the WM performance of individuals with hearing impairment was slower in comparison to better-hearing individuals during the A-only condition, no group differences were observed during the AV condition. Thus, it appears that visual speech cues may help to counteract the slowing of information processing caused by hearing impairment.

Surprisingly, no difference between the AV and the A-only condition was evident in the accuracy data suggesting that despite the facilitation of processing speed, the AV speech did not seem to influence overall WM capacity. There was also no effect of group on accuracy. Overall, these results indicate that both older adults with hearing impairment and those with age-normal hearing are able to achieve similar levels of accuracy during A-only and AV speech, however they are able to achieve these levels of accuracy at faster RTs when visual speech cues are available.

On electrophysiological correlates of WM, facilitation of processing speed (indicated by P3 latency) was observed in both groups and facilitation of WM resources (indicated by P3 amplitude) was observed in the individuals with hearing impairment. More specifically, both older adults with hearing impairment and those with age-normal hearing showed earlier P3 latency in the AV condition compared to the A-only condition, further validating the finding of improved processing speed during AV speech observed in the behavioral RT data. Overall, there seems to be a disproportionate gain on WM processing speed when perceptual processing speed is facilitated. That is, the average facilitation of P1 latency was 5.0 ms (*SD* = 11.33) and N1 latency was 3.8 ms (*SD* = 18.28) whereas the average facilitation was 35.5 ms (*SD* = 53.40) for P3 latency and 82.6 ms (*SD* = 53.35) for RT. In addition, we observed that P3 amplitude was smaller during the A-only condition in hearing-impaired participants compared to controls but no group differences were evident in the AV condition. Thus, similar to the RT data, it appears that visual speech cues may help to counteract the negative effect of more demanding perceptual processing caused by hearing impairment.

### Do older adults with hearing impairment show a greater auditory-visual speech benefit?

The results of this study have confirmed that perceptual processing was more demanding for older adults with hearing impairment. This was suggested by a significantly smaller amplitude of the auditory P1-N1 component in older adults with hearing impairment compared to better-hearing controls. N1 amplitude is known to be affected by stimuli characteristics, such as intensity and tonal frequency (Näätänen and Picton, 1987). Thus, it appears that physically similar stimuli become “tuned down” and less perceptible in the context of hearing impairment. Furthermore, there was a statistical trend for a delayed auditory N1 latency in the older adults with hearing impairment in comparison to the better-hearing controls, suggesting prolonged perceptual processing time. These results agree with the finding of Oates et al. (2002) who found an increased latency and a decreased N1 amplitude with increasing hearing loss during a syllable discrimination task. In contrast, studies using more ambiguous stimuli during speech discrimination tasks, found increased N1 amplitudes in individuals with hearing impairment (Tremblay et al., 2003; Harkrider et al., 2006). In the current study, the effects of hearing impairment were also evident on WM measures. Older adults with hearing impairment had smaller P3 amplitude and longer RT during the A-only condition compared to the control group. In addition, the group with hearing impairment had generally greater P3 latency, regardless of modality.

When comparing the overall results between better-hearing older adults and those with hearing impairment, the pattern suggests that older adults with hearing impairment are deriving a greater AV speech benefit than better-hearing older adults. Firstly, older adults with hearing impairment showed greater facilitation of perceptual processing, as evidenced by the greater reduction in P1 and N1 latency and P1 and P1-N1 amplitudes in the AV condition compared to the A-only condition. Furthermore, both behavioral RT data and electrophysiological P3 amplitude data suggest greater facilitation of WM processing in older adults with hearing impairment. More specifically, the group differences were observed in the baseline (i.e., A-only) condition but not during the AV condition, indicating that visual speech cues helped older adults with hearing impairment to compensate for the difficulty that they experienced during the more demanding A-only condition. The observed findings are in agreement with previous behavioral research reporting improved speech recognition under AV conditions in individuals with hearing impairment (Grant et al., 1998; Tye-Murray et al., 2007; Bernstein and Grant, 2009). Furthermore, these results support the indication of greater AV benefit in older adults with hearing impairment compared to those with better hearing observed in a syllable recognition paradigm by Grant et al. (2007) as well as in a behavioral WM paradigm by Brault et al. (2010). Overall, the greater AV benefit in older adults with hearing impairment supports the inverse-effectiveness hypothesis, which proposes that the benefit from multisensory interaction increases as the functioning of unisensory channels decreases (Stein and Meredith, 1993).

When examining the direct relationship with correlation analyses between perceptual facilitation (i.e., facilitation of P1-N1 amplitude and N1 latency during the AV condition in comparison to A-only condition) and behavioral improvement (i.e., higher accuracy and faster RT in the AV in comparison to A-only condition), we found that better-hearing older adults showed a reliable dose-response relationship between these variables, especially for facilitation of processing speed. A reliable relationship was found between greater facilitation of N1 latency in the AV condition compared to the A-only condition and greater improvement in RT during the 2-back condition. Similar trends were observed across other conditions. Interestingly, the BH group did not show a reliable AV benefit for neither N1 latency nor P1-N1 amplitude in the group ANOVA analyses. Taken together these results suggest that even though older adults with better hearing may have shown more inconsistent perceptual facilitation as a group (i.e., in the ANOVAs), those who derived a perceptual benefit from the AV speech were also able to benefit at the WM level, especially in terms of facilitation of processing speed (as demonstrated by the correlation analyses). One might question why a reliable relationship was only demonstrated between the N1 latency and 2-back RT performance. We would argue that this finding shows a relationship between two logically similar measures of processing speed in the experimental condition that was most demanding of WM resources. One might not expect reliable relationships between more conceptually dissimilar measures (e.g., ERP amplitude vs. RT; or at levels of non-demanding working memory load). Moreover, the behavioral measures represent the output of a number of preceding processes, including sensory and perceptual processing, working memory operations, response biases, and decision making thresholds, while the ERPs can be taken to be more discrete and temporally specific. Nevertheless, we should be cautious in our interpretation of these correlational findings and we encourage replication with a larger independent sample of participants.

On the other hand, participants with hearing impairment showed a more robust perceptual AV benefit, as indicated by facilitation of N1 latency in the 1-back condition and overall facilitation of P1-N1 amplitude evident in the group ANOVAs, but were found to have a less clear dose-response relationship between perceptual facilitation and WM performance (i.e., the correlation analyses). This may be related to the fact that perceptual facilitation helps individuals to reach their WM capacity but not necessarily to expand its limits. Thus, participants may gain a variable level of perceptual facilitation but, regardless of this variability, may achieve similar improvement on behavioral measures. This hypothesis is supported by the observation that no behavioral differences were observed between the groups in the AV condition. For reaction time specifically, individuals with hearing impairment were slower in comparison to controls in the A-only condition but not in the AV condition. Thus, visual speech cues appeared to improve their WM capacity to the point that their performance no longer differed from those with better hearing.

### Practical implications

The statistics clearly highlight the high prevalence of social and psychological difficulties in the hearing impaired population (e.g., Strawbridge et al., 2000; Dalton et al., 2003). AV speech represents one possibility for facilitation of information processing and thus improved communication abilities for older adults with hearing impairment. Furthermore, numerous speech comprehension training programs have been developed over the years (see Pichora-Fuller and Levitt, 2012) and previous research has found that speech-reading training can improve speech perception of individuals with hearing impairment (e.g., Walden et al., 1981; Richie and Kewley-Port, 2008). The results of the current study indicate that such training may be beneficial not only for enhancement of perceptual but also for higher-order functioning.

In addition to speech comprehension training, the current results have implications for technology adaptation and future development. For example, despite their increased popularity in commercial companies and government institutions, research has shown that older adults find it very challenging to use interactive voice response (IVR) services (Miller et al., 2013). Capitalization on AV speech may provide one method for making future technology user-friendlier for older adults, especially those with hearing impairment.

### Limitations

Several methodological and statistical limitations of the current study need to be acknowledged. Firstly, a larger sample size would decrease error variance and provide greater statistical power. In a previous study with a similar design (Frtusova et al., 2013) but a greater sample size, we found AV facilitation of both N1 latency and P1-N1 amplitude in older adults with age-normal hearing. In the current study, the modality effect on these perceptual measures did not reach statistical significance for this group even though the means pointed in the right direction (see Tables 2, 5). Secondly, a consideration needs to be given to our sample of older adults with hearing impairment. Individuals in the hearing impaired group were quite heterogeneous in terms of their level of hearing impairment (average PTA ranging from 31.67 to 73.33 dB), and their general cognitive ability as estimated by the MoCA (overall score ranging from 21 to 30 points). However, exploratory analyses showed that these factors were not systematically associated with the level of AV benefit. On the other hand, a significant correlation between higher contrast sensitivity and a lower AV benefit on P1-N1 amplitude was observed for the 1-back (*r* = −0.49) and the 2-back (*r* = −0.48) conditions. Thus, those older adults with hearing impairment who also have poorer visual ability seem to derive the largest AV benefit. Another consideration is that we were unable to confirm for all the participants the exact nature of their hearing impairment; some participants were unsure of the cause and did not have an audiology report available. Nevertheless, all participants reported wearing or being eligible to wear hearing aids, which is most commonly prescribed for older adults with sensorineural hearing loss. Lastly, information about the exact length of hearing aid use was not available for all participants, which may obscure heterogeneity in this group in regard to any potential disadvantage when being tested without a hearing aid.

### Conclusions

This study provides evidence that older adults derive WM benefit from AV speech. Importantly, these effects were found to be even more robust in older adults with hearing impairment compared to those with better hearing. In the context of an integrated perceptual-cognitive system, these results indicate that AV speech facilitates perceptual processing that is otherwise very demanding for older adults with hearing impairment. The perceptual facilitation results in more resources available for subsequent WM processing. The evidence of processing facilitation afforded by AV speech has important practical implications for helping to improve the quality of life for older adults with hearing impairment.

## Author contributions

JF contributed to the conceptualization of the study and study design as well as designed the stimuli and experimental paradigm. She also contributed to the acquisition, analysis and interpretation of the data and wrote the draft of the study. She approved the final version and agreed to be accountable for all aspects of the work in ensuring that questions related to the accuracy or integrity of any part of the work are appropriately investigated and resolved. She completed the study as a part of her Doctoral Dissertation. NP contributed to the conceptualization of the study and study design as well as analysis and interpretation of the data. She also revised the draft of the manuscript and critically evaluated it for important intellectual content. She approved the final version and agreed to be accountable for all aspects of the work in ensuring that questions related to the accuracy or integrity of any part of the work are appropriately investigated and resolved.

## Funding

This research was conducted in the absence of any commercial or financial relationships that could be construed as a potential conflict of interest. The study was supported by grants awarded to NP from the Canadian Institutes of Health Research (Grant MOP-97808) and the Concordia University VPRGS Seed/Accelerator Funding Program (2009). We would like to thank to the Alzheimer Society of Canada for Alzheimer Society Research Program Doctoral Award awarded to JF. We would also like to express gratitude to the Centre for Research in Human Development for the financial and technical support to the Cognition, Aging, and Psychophysiology Lab where the research was conducted. Finally, we would like to thank the members of the Cognition, Aging, and Psychophysiology Lab for their contribution to the project. Special thanks to Ms. Lianne Trigiani for her help with data processing and to Ms. Mariya Budanova for her help with testing and data processing.

### Conflict of interest statement

The authors declare that the research was conducted in the absence of any commercial or financial relationships that could be construed as a potential conflict of interest.
